# Monitoring oil spill contamination in seawater using molecularly imprinted polymer thin-films and direct solid-state fluorometric detection

**DOI:** 10.1007/s10661-026-15841-w

**Published:** 2026-08-27

**Authors:** Hassan Y. Hijazi, Maryam Tahir, Safa Salim

**Affiliations:** 1 College of General Education, Sciences, Chemistry, University of Doha for Science and Technology, Doha, Qatar; 2 College of Engineering and Technology, Chemical Engineering, University of Doha for Science and Technology, Doha, Qatar

**Keywords:** Oil spill, Dibenzothiophene, Molecularly imprinted polymer, Thin films, Seawater, Fluorometric detection

## Abstract

A molecularly imprinted polymer thin film (MIP) on a polyethylene terephthalate (PET) substrate for the selective extraction and solid-state fluorescence detection of dibenzothiophene (DBT) as a marker of oil spills in marine environments was developed. The MIP thin films were produced via UV-initiated polymerization of a polymeric mixture, giving a mechanically robust thin film without the necessity to modify the substrate surface. This solid-state fluorometric technique eliminated the need for extraction by providing a direct measurement of fluorescence from the solid phase, reducing sample pre-treatment requirements. Linear responses were observed over a concentration range of 5.0 to 80.0 µg L⁻^1^ (*R*^2^ = 0.9906). Furthermore, a detection limit of 1.74 µg L⁻^1^ (*n* = 15) was established. Acceptable selectivity towards DBT was shown by the MIP thin films in comparison to structurally similar aromatic compounds such as naphthalene and indole, which resulted in minimal interference with the signal. The reliability of the thin-film was further proven when the film was applied to untreated seawater samples, resulting in negligible matrix effects and quantitative results. In addition, the flexibility of the PET substrate will allow an easy adaptation to portable sensing units. This new MIP thin-film with rapid fluorescent detection offers a highly sensitive, selective and user-friendly tool for the early detection and assessment of oil spills in marine environments.

## Introduction

Oil spills continue to cause major problems with marine pollution, causing ecological harm to aquatic ecosystems, impacts on the coastal environment, and affecting social/economic activities which use marine resources (Liu et al., 2026). Hydrocarbons derived from petroleum can last for years in the water and sediment of the ocean and affect the process of exchanging oxygen into the water body, and will inhibit photosynthesis, as well as create toxic conditions for marine life forms (Perhar & Arhonditsis, 2014). The extent of the environmental impact caused by oil contamination will greatly depend on how quickly and accurately the contamination can be detected. Early detection will allow for rapid responses to prevent additional ecological damage and save money on remedial work (X. Li et al., 2024). Therefore, developing reliable monitoring devices that can detect very small amounts of petroleum-derived chemicals in marine environments continues to be an essential goal in conducting environmental monitoring research (Beyer et al., 2016; Phillips & Rainbow, 1993; Sun et al., 2026). The primary means for oil spill monitoring are remote sensing technology, laboratory-based chromatographic technology and fluorescence-based detection technology. Remote sensing technologies can monitor large areas of land or water, but can be impacted negatively by weather conditions, wave action and the quality of light reaching the sensors. Chromatographic techniques using instruments such as gas chromatography-mass spectrometry (GC–MS) provide excellent specificity and accuracy in analyzing samples collected from an oil spill site. However, collecting samples, transporting them back to a lab, preparing them for analysis and waiting for results takes too much time to enable rapid assessment of the level of environmental damage. Fluorescent spectroscopy has become a viable option for rapidly determining levels of aromatic hydrocarbons in water since many petroleum components fluoresce when exposed to UV radiation (Dhavalikar & Choudhari, 2022; Gaines et al., 1999; Poudel et al., 2025; Prince & Walters, 2022; Wang & Fingas, 1997). Dibenzothiophene (DBT) is a heterocyclic sulfur containing aromatic compound found in most crude oils and petroleum products. Due to its high chemical stability and resistance to biodegradation, DBT persists in marine environments longer than many volatile hydrocarbons. DBT has been identified as being directly related to petrogenic contamination. Therefore, DBT is considered a molecular marker for determining if petroleum contamination exists in seawater. Additionally, DBT exhibits unique fluorescent properties upon excitation with UV light, enabling its detection at low concentrations (Wang & Fingas, 1997; Berthou & Vignier, 1986; M. Li et al., 2012). As a result, DBT is considered an ideal model compound for assessing various analytical techniques used for detecting petroleum-derived contaminants in marine systems.

Molecularly imprinted polymers (MIPs) have emerged as promising recognition materials for environmental monitoring because they combine antibody-like molecular selectivity with excellent chemical stability, low production cost, and long-term durability under harsh environmental conditions. Since MIPs can selectively extract pesticides, pharmaceuticals, endocrine disruptors, petroleum-derived hydrocarbons, etc., from aqueous solutions, this technique has attracted significant attention as a method for detecting a wide range of contaminants found within our environment (Belbruno, 2018; Gkika et al., 2024; Tian et al., 2026). The most recent advancements in molecular imprinting techniques have also enabled the development of highly selective functional materials and bio-mimetic sensor surfaces for use in environmental and bio-analytical applications (Kadhem et al., 2021; Ramajayam et al., 2023). Although there are several advantages associated with the development of MIP-based sensing platforms for the direct analysis of complex environmental samples, this development remains challenging due to dissolved organic matter, ionic interference, and structural similarity between analytes and interferences, which result in reduced selectivity and increased matrix effects (Arabi et al., 2025). In addition, much of the previously published literature describing MIP-based analytical techniques required additional steps beyond adsorption to include extraction, desorption, and/or chromatography. As such, the ability to perform rapid analyses directly in the field is limited (X. Arabi et al., 2021; Li et al., 2024).

The use of fluorescence has made analytical techniques based on fluorescence very fast and very sensitive to detecting aromatic compounds derived from petroleum. The sensitivity and speed associated with using fluorescence make it a good candidate for environmental monitoring. Conventional methods of fluorescence measurement used to measure seawater are often hampered due to spectral interference caused by dissolved organic material and naturally occurring fluorophores. This is a problem because it reduces the specific nature of analytical results. Selectively recognizing and concentrating target analytes at the sensing interface, while also minimizing non-specific adsorption and spectral interference from the sample matrix, can be accomplished by integrating MIPs as selective recognition films. Still, there are a limited number of studies that describe flexible thin-film MIP platforms that allow for the solid-state fluorescence analysis of untreated seawater samples. Direct analysis of untreated seawater will be a crucial goal for monitoring environmental pollution since the use of filters, extractants, and similar sample treatment methods to prepare samples can extend the time required for analysis, limit the availability of laboratory equipment, and potentially modify both the distribution and quantity of petroleum-derived pollutants in the sample. Therefore, the development of analytical systems capable of directly analyzing untreated seawater provides the ability to make decisions regarding oil spills quickly, as well as reduces the number of possible sources of error associated with manipulating the sample. The recent growth of research into MIP-based sensor technology demonstrates increasing interest in developing materials that exhibit high selectivity and good analytical performance in environmentally complicated conditions, without the need for extensive sample preparation (X. Arabi et al., 2025; Li et al., 2024). Additionally, eliminating extraction procedures that rely on solvents is also in line with the principles of green chemistry for analytical purposes (Green Analytical Chemistry), since it results in reduced solvent usage, reduced waste from chemicals generated during the process of extraction and ultimately facilitates the entire analytical process (Gałuszka et al., 2013).

The overall objective of this research was to develop and evaluate an MIP thin-film for the selective fluorometric determination of DBT directly in seawater as an indicator of petroleum contamination. Although MIPs have been widely investigated as selective adsorbents and sensing materials for environmental pollutants, most reported MIP-based analytical methods require analyte extraction, desorption, or chromatographic analysis after adsorption. In contrast, the present work integrates selective molecular recognition with direct solid-state fluorescence detection using a flexible polyethylene terephthalate (PET)-supported MIP thin-film, enabling DBT to be measured directly while retained within the imprinted polymer. This analytical strategy eliminates extraction and pre-concentration steps, reduces sample handling and analysis time, and enables direct analysis of untreated seawater with minimal matrix interference. Consequently, the proposed sensing platform provides a practical combination of selectivity, analytical simplicity, portability, and green analytical characteristics that make it attractive for rapid environmental monitoring of petroleum contamination.

## Materials and methods

### Chemicals and reagents

Dibenzothiophene (DBT, ≥ 99%), 2-thiophenecarboxaldehyde (2-TCA, 98%), naphthalene (99%), indole, 1-vinylimidazole (1-VIM, ≥ 99%), ethylene glycol dimethacrylate (EGDMA, 98%), and 2,2-dimethoxy-2-phenylacetophenone (DMPA, 99%) were all purchased from Sigma-Aldrich. Methanol, hexane, 1-octanol, acetone and acetonitrile were ACS reagent grade and purchased from ACP Chemicals. Polyethylene terephthalate (PET) sheets (30 cm × 30 cm) were purchased from a local store in Doha, Qatar, and micro cover glasses (18 × 18 mm) were purchased from Fisher Scientific. 20 mL glass vials from Agilent Technologies, USA. Deionized (DI) water (18 MΩ cm) was produced by a water purification system (Barnstead Nanopure Water Systems, Lake Balboa, USA).

### Pre-treatment and preparation of PET substrates

To prepare the PET substrate slides for application of an MIP thin-film, the PET sheets were cut into 25 × 25 mm square slides, and then a series of sequential cleaning steps was applied to the surfaces of the slides. The first cleaning step involved washing the slides with laboratory detergent and thoroughly rinsing the slides with DI water to remove all dust, grease, etc. Cleaning with detergent/water also removes any soluble residue that may be left on the slide. Following this initial cleaning, the slides are again rinsed with methanol to further remove any other organic compounds/impurities that remain after the first wash. Additionally, methanol will help to replace any residual water that has remained on the surface due to the previous washing steps, thus enhancing both wetting and subsequent adhesive properties between the polymer matrix and the substrate during the polymerization process. Finally, a final rinse is done with acetone to ensure that no additional contaminants remain on the surface. After all contaminants had been removed from the slides, they were dried with a slow flow of dry nitrogen and stored for later use. Drying under nitrogen prevents potential contamination of the slides when exposed to ambient air. Solvent evaporation occurred rapidly, and there was little chance that solvent residuals would remain on the surface of the slides.

### Preparation of MIP thin-film slides

Preparation of the MIP thin-film was based on a modified version of a previous method (Hijazi & Bottaro, 2020). Pre-polymerization reaction was performed by mixing 16.3 µL (0.178 mmol) of template (2-TCA), 64.6 µL (0.713 mmol) of monomer (1-VIM), 672.7 µL (3.57 mmol) of cross-linker (EGDMA), 14.3 mg (0.06 mmol) of photoinitiator (DMPA) and 1000 µL of solvent (1-octanol) in a 4 mL glass vial; all chemicals were transferred either by weighing or pipetting. The dummy template 2-TCA was used in this study due to the presence of an aromatic heterocyclic containing sulfur which is also present in the molecule DBT, thereby responsible for its major molecular recognition interactions. In a previous study, we successfully employed and validated this strategy (Hijazi & Bottaro, 2020), where selective molecular recognition cavities were formed for sulfur containing aromatic molecules. Using a structural analogue as a dummy template is a widely accepted method of forming molecular imprints, which can be effective at minimizing bleed from templates and providing reliable results in trace-level analyses (Belbruno, 2018; Chen et al., 2004). The effectiveness of this approach is shown through the high imprinting factor, selective recognition of DBT over structurally related aromatic compounds, and successful application to untreated seawater samples, confirming that the recognition cavities generated using 2-TCA effectively recognize DBT. Then, the mixture underwent an ultrasonication in a water bath for 10 min to remove dissolved oxygen that could interfere with subsequent photo-initiated polymerization. Subsequently, 8 µL of prepolymerization mixture was pipetted on the surface of a PET slide (25 × 25 mm); at once, a glass microcoverslip (18 × 18 mm) was placed over the drop. Upon even spreading of the droplet, it was exposed to UV radiation (365 nm) for 40 min. Following this, the cover slide was carefully lifted off, and the slide coated with the MIP thin-film was washed in methanol solutions to remove template molecules and unreacted components. Methanol solution was changed every hour; three hours later, the washing time was stopped as there was no significant increase in fluorescence intensity, indicating complete release of unreacted components from the polymer film. To ensure reproducible fabrication, the deposition volume (8 µL), the dimensions of the PET substrates and cover glasses, and the conditions for UV polymerization were kept constant across all preparations. The selected deposition volume consistently produced a uniform, continuous and mechanically stable thin-film of MIP that completely covers the sensing area and produces reproducible fluorescence responses. Thus, fabrication geometry was controlled by standard fabrication conditions while porogen volume was systematically varied to optimize performance and recognition of molecules in thin-films. This procedure provided a stable background for fluorescence measurements in the solid-state and reduced interference due to emission from the polymer matrix itself. Background fluorescence of the polymer films was measured to verify negligible interference from the polymer matrix. A non-imprinted polymer (NIP) film was synthesized and washed similarly, but without adding template molecules. The mass of film (m_film_, ~ 3 mg) was calculated by subtracting the mass of the MIP slide before polymerization from its mass with the deposited film. An analytical balance with an accuracy of ± 0.01 mg was used to measure the film masses. The imprinting factor (IF) was evaluated by comparison of the binding capacities of MIP thin-films and NIP films for DBT under equal experimental conditions. The binding capacity (Q) was determined from the subtraction of the initial equilibrium concentration of DBT in solution multiplied by the sample volume and normalized to the mass of the MIP thin-film used. To assess the reproducibility of the MIP film fabrication process, three independent preparations were made from the same optimal thin-film formulation; all three samples used the same amounts of reagents, as well as the same polymerization process. The films showed a reproducible fluorescence response to the analyte, with the relative standard deviations (RSDs) summarized in Table 1.

**Table 1:** Imprinting factors (IF) values at various 1-Octanol (porogen) volumes

1-Octanol Porogen Volume	1-Vinylimidazole (1-VIM) Mass and Volume	Ethylene Glycol Dimethacrylate (EGDMA) Mass and Volume	Mass of MIP thin-Film on PET Substrate	Imprinting Factor (IF) (RSD, *n* = 3)
800 µL	67.1 mg (64.6 µL)	707.0 mg (672.7 µL)	-	Film cracked, IF could not be determined
1000 µL			2.86 mg	2.21 (3.4%)
1200 µL			3.23 mg	2.76 (8.2%)
1400 µL			2.31 mg	1.65 (11.6%)

### Fluorescence measurements

A PerkinElmer LS-45 fluorescence spectrofluorometer, utilizing a xenon light source as well as a solid sample holder to measure the solid film samples that were used to perform fluorometry. Fluorescence at different angles (5°–60°) of the MIP thin-film was measured by positioning the solid holder such that the light source (excitation beam) was concentrated into a very small area (1 × 5 mm) on the MIP thin-film surface. In an effort to minimize the amount of background fluorescence from the MIP blank network, the individual components used to create the MIP thin-films were chosen based upon their low levels of background fluorescence. The following parameters were established for the fluorometric measurements: excitation wavelengths: from 200 to 480 nm with a sampling interval of 5 nm; emission wavelengths: from 300 to 500 nm with a sampling interval of 5 nm; slit width for excitation wavelength: set to 10 nm; slit width for emission wavelength: set to 10 nm; integration time: set to 0.50 s; photomultiplier tube voltage: set to Auto Voltage. Upon performing these measurements, it was determined that the excitation wavelength for measuring the MIP thin-films was found to be 290 nm, and the corresponding emission wavelength is 343 nm. Therefore, both wavelengths were utilized for all subsequent fluorescent measurements.

### Seawater samples collection

Seawater samples were collected from various sites around Doha harbor in Qatar by collecting four-litre quantities of pre-cleaned amber glass bottles. Prior to adding the sample, each bottle was thoroughly cleaned and rinsed with the sampled water. Each bottle was then filled entirely and tightly closed with a cap that was sealed with paraffin tape. Bottles containing the samples were then stored under refrigeration (~ 4 °C) and analyzed as soon as possible after collection; therefore, they did not require preservation agents such as chemical preservatives to protect their natural state. The pH of the collected seawater samples was approximately 8.0 ± 0.3 at room temperature. No adjustments to the pH of the samples were made before analysis, to provide an assessment of how well the developed MIP thin-film would perform under natural seawater conditions. Aliquots of 20 mL of raw seawater samples were placed into clean glass vials and spiked with DBT at concentrations of 5.0, 10.0, 20.0, 40.0, 60.0, or 80.0 µg L^−1^, respectively, with three replicates for each concentration. Thin-film coated MIP-slides were then placed in contact with each spiked sample and allowed to equilibrate for one hour while being agitated on a magnetic stirrer at 500 rpm. Following the equilibration time, the MIP-slides were removed and washed lightly with deionized water to remove loosely associated compounds. Each slide was permitted to dry in air for five minutes and then affixed directly to the solid sample holder of a fluorimeter under optimal operating conditions. Fluorescence was measured immediately upon mounting the MIP thin-film slides at optimal excitation and emission wavelengths.

### Selectivity experiment

The selectivity of MIP thin-films towards DBT was studied in both DI water and seawater with structurally similar aromatic compounds that could potentially interfere, such as naphthalene and indole. In the first series of tests, the MIP thin-films were placed into 20 mL glass vials with DI water that had been spiked with 40 µg L^−1^ of DBT and different amounts of naphthalene (i.e., 0, 10, 20, and 40 µg L^−1^). The test protocol was followed with the use of indole instead of naphthalene, while maintaining the same concentration ranges (0, 10, 20, and 40 µg L^−1^), so that it can be determined whether indole will cause an interference effect. Additionally, experiments involving simultaneous interference from naphthalene and indole were performed in DI water and seawater, which contained 40 µg L^−1^ DBT, 40 µg L^−1^ naphthalene and 40 µg L^−1^ indole to study the effects of multiple interferents on the performance of MIP thin-films under conditions representative of environmental contamination. In all cases, after being submerged in the spiked solution for one hour with constant agitation via a magnetic stirrer, the MIP thin-films were carefully removed, rinsed with a small volume of DI water to remove weakly bound species, air-dried, and then mounted on the solid sample holder of the fluorometer for measurement. Each sample was conducted in triplicate. The relative fluorescence response (F/F₀) was calculated as the ratio between the fluorescence intensity measured in the presence of DBT together with the interfering compound (F) and the fluorescence intensity measured for the corresponding DBT solution without any interferent (F₀).

## Results and discussion

### MIP thin-film formation and adhesion

MIP thin-films were developed using a method previously published (Hijazi & Bottaro, 2020). Solid white thin-films produced with a good appearance were formed by sandwich techniques in an 18 × 18-mm size on a PET substrate under UV photopolymerization conditions. Covalent grafting between the MIP thin-film and the PET substrate was not observed. As there was no need for PET substrate surface pretreatment in our work, the MIP thin-film produced appeared to exhibit sufficient adhesion to the surface of the PET substrate during in situ polymerization, which may be due to the intimate contact formed at the interface and interfacial interactions between the polymer network and the substrate. While surface treatments such as plasma treatment or chemical modification could be used to improve adhesion, it was not necessary since the films did not degrade after being subjected to repeated washing and fluorescence measurements, as indicated by the thin-film remaining firmly attached without delamination or tearing occurring. PET substrates offer many advantages over glass when used for applications related to MIP thin-films. They are lightweight, flexible, and mechanically robust, thus reducing the risk of breakage and making them easy to handle and deploy in portable and field-based sensing platforms. For this application, the PET substrates only require simple pretreatment (cleaning) prior to use. Typically, glass substrates require additional surface modification, such as silanization, to improve film adhesion. However, avoidance of these extra steps reduces chemical use and organic solvent use, simplifies fabrication processes, and increases cost-effectiveness and environmental compatibility. These characteristics make PET substrates more suitable for integration into practical sensing and detection systems.

The selection of MIP components was carefully made. Intrinsically low fluorescence emission from the MIP components is essential when measuring the fluorescence signal directly from the solid thin-film after exposure to seawater samples. Because the analytical signal originates from the fluorescence of the target analyte (DBT) immobilized within the imprinted cavities, any background fluorescence from the polymer matrix, functional monomer, crosslinker, or initiator may contribute to baseline noise, spectral overlap or inner filter effects, resulting in reduced sensitivity and selectivity (Yan et al., 2024). This becomes particularly important in solid-state fluorescence measurements where the polymer film itself is directly mounted on the fluorometer without prior extraction of the analyte. Additionally, naturally occurring organic matter and other background fluorescence present in seawater matrices can further complicate the interpretation of signals. Therefore, selection of MIP components exhibiting minimal intrinsic fluorescence in the excitation-emission region of DBT helps ensure a low baseline signal, improves signal-to-noise ratio and enhances reliability of quantitative measurements. In addition, minimization of polymer-related fluorescence also reduces the risk of spectral interference and facilitates accurate assessment of selective binding. As a result, the MIP thin-film is more suitable for direct solid-phase fluorescence sensing applications in complex environmental samples like seawater. Therefore, the functional monomer 1-VIM and the crosslinker EGDMA were selected based on their relatively low fluorescence emission in the spectral region of DBT. In addition, the use of 1-octanol as a porogen results in the formation of a porous structure without significant background fluorescence. While Bisphenol A dimethacrylate (BPADMA) has been shown to produce better adsorption properties than EGDMA in our previous work (Hijazi & Bottaro, 2020), it was not selected for use in this study since its intrinsic fluorescence will likely overlap with the DBT emission signal, reducing detection sensitivity in solid-state measurements.

In our previous work (Hijazi & Bottaro, 2020), the MIP thin-film was characterized, as well as the optimization of adsorption studies (including adsorption isotherms and adsorption kinetics). However, this current investigation focused on optimization of the film formulation through examination of the influence of the volume of 1-octanol porogen upon the IF and the ability to improve reproducibility of the fluorescence response measured directly from the solid MIP thin-film. Octanol was used as the porogen due to its low vapor pressure properties and potential to form both macro- and mesopores within the film (Zhang et al., 2009). The volume ratio of the porogen affects the degree of porosity of the polymer, the accessibility of binding sites and the surface area available for adsorption. Therefore, as seen in Table 1, the effect of porogen concentration upon the adsorption characteristics of the MIP thin-film was examined through varying porogen volume while maintaining constant ratios of T:M:C (1:4:20) and total mass of monomers and cross-linkers.

Values in Table 1 are presented as the means of three independent thin-films preparations. The Relative standard deviations (RSDs) are shown in parentheses and were calculated from triplicate measurements. The imprinting factor (IF) is defined as the ratio of the binding capacity of an MIP to that of its corresponding NIP under identical experimental conditions. No reliable mass or IF values could be obtained for films made with a formulation containing 800 µl of 1-octanol due to brittleness and cracking. All formulations had a template: monomer: cross-linker mole ratios of 1: 4: 20; variation was only in the porogen volume.

We found that the use of 800 μL resulted in fragile and cracked films. The highest IF was achieved using an MIP thin-film that was made using 1200 µL of porogen (Table 1). However, this film had low reproducibility compared to the film prepared using 1000 µL, since its relative standard deviation (RSD) was much higher. It is also interesting to note that increasing the porogen volume beyond 1200 µl significantly decreased both the IF and reproducibility of the film. The porogen volume ratio not only affects the morphology of the MIP but also has a vital role in its IFs and reproducibility (Lah et al., 2021). As a result, 1000 µL was chosen as the optimal porogen volume for final film preparation.

### Evaluation of MIP thin-film fluorescence and sensitivity

**Fig. 1:**
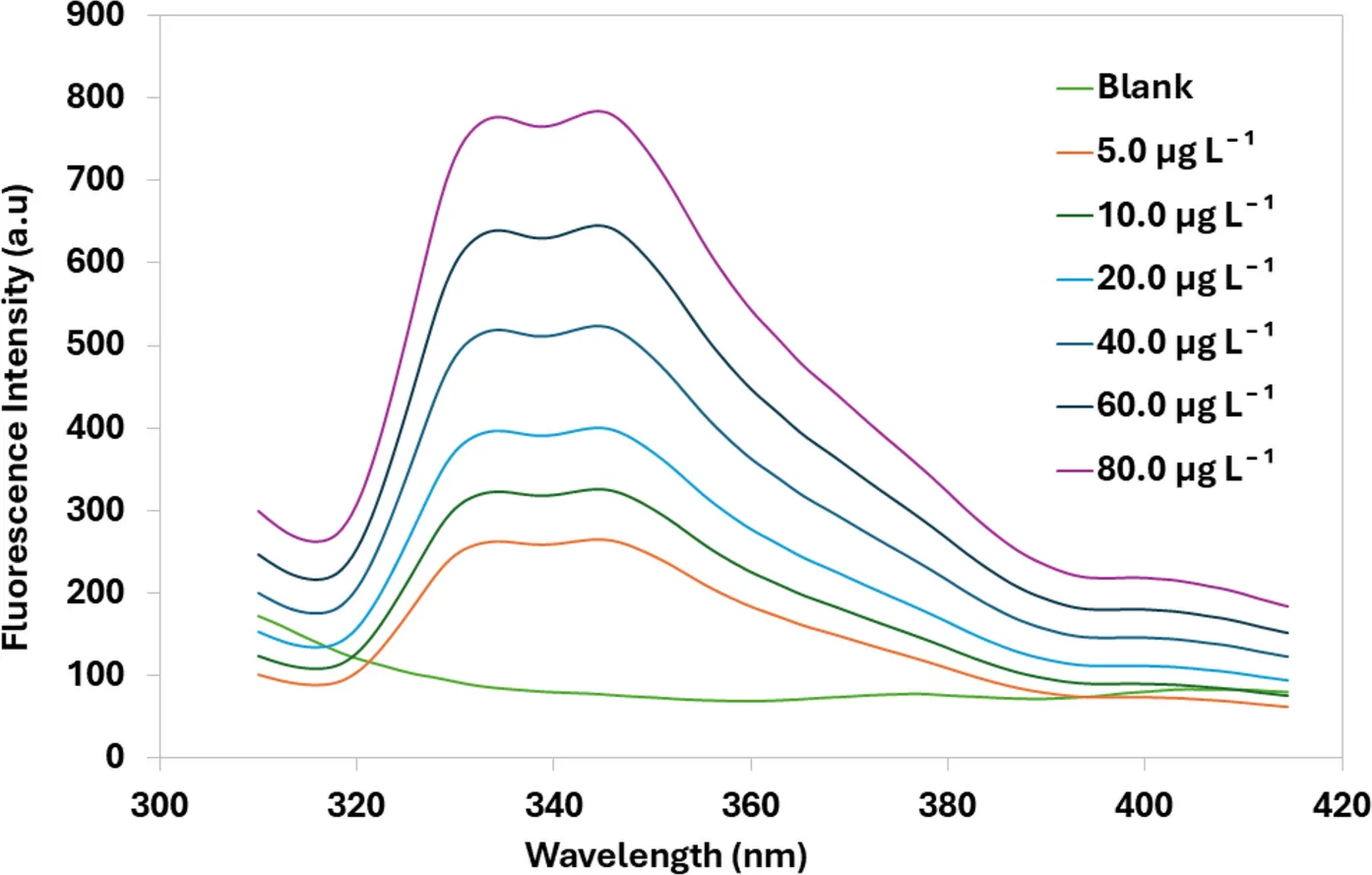
Fluorescence emission spectra of MIP thin-film at varying DBT concentrations

Fluorescence experiments were performed with a fluorometer, employing a solid-sample support system, so that it is possible to directly analyze the DBT-MIP thin-films after uploading to a spiked DI-water or seawater solution after a gentle rinse with DI water. The advantages of this direct solid-state technique include the avoidance of analyte extraction and the examination of the fluorescence response generated from DBT molecules adsorbed into the imprinted cavities of the thin-film. The excitation and emission wavelengths found for DBT-MIP films were 290 nm and 343 nm, respectively. These values were selected for all subsequent fluorescence measurements due to their higher sensitivity and lower backgrounds from the polymeric matrix compared to other available combinations. To optimize the measurement parameters prior to performing calibration studies, an investigation of the influence of detector geometry on fluorescence intensity was performed. Emission responses of MIP thin-films previously exposed to DI water containing 40 μg L^−1^ DBT were examined over a range of detection angles (from 5° to 60°). It was demonstrated that fluorescence intensity increases by increasing the detection angle to approximately 25°, beyond which point, a gradual decrease in fluorescence signal occurs. Therefore, a detection angle of 25° was chosen for all subsequent measurements because it produced the maximum fluorescence response. The fluorescence response of MIP thin-film towards DBT was studied by exposing films to DI water solutions containing DBT at concentrations of 5.0, 10.0, 20.0, 40.0, 60.0, and 80.0 μg L^−1^. In addition, a blank MIP thin-film was measured under identical conditions as those used to study the DBT-spiked DI water solutions to assess background fluorescence contributions from the polymeric matrix. Results illustrated in Fig. 1 show that the blank film exhibits very low fluorescence intensity throughout the scanned wavelength range, indicating that the selected polymeric components have negligible intrinsic fluorescence and will not significantly interfere with DBT detection. The ability to distinguish clearly between the blank and DBT-MIP thin-films further supports that the measured fluorescence is primarily from DBT molecules that are selectively absorbed into the imprinted cavities of the polymer.

When MIP thin-films were exposed to DBT solutions, there was a significant increase in fluorescence intensity, characterized by emission at 343 nm. Furthermore, as DBT concentrations increased, there was an increase in fluorescence intensity, illustrating that the MIP thin-film responds in a concentration-dependent manner.

This is indicative of an effective adsorption of DBT molecules within the imprinted polymer matrix, which has created a microenvironment that has allowed for an improved fluorescent response. Our previous research has investigated both the influence of non-selective interactions with a polymer matrix and the function of template-functional monomer interactions in the determination of the specificity of molecular recognition by films prepared via this method (Hijazi & Bottaro, 2020). Therefore, we focus on improving film performance and analytical response based on the understanding of the mechanisms of recognition previously developed. A significant progression in the fluorescence response was seen through the DBT concentration ranges (from 5.0 to 80.0 µg L^−1^), demonstrating a high level of sensitivity of the MIP thin-film over the investigated concentration range and supporting the potential of the developed solid-state fluorescence method for detecting low levels of DBT.

**Fig. 2:**
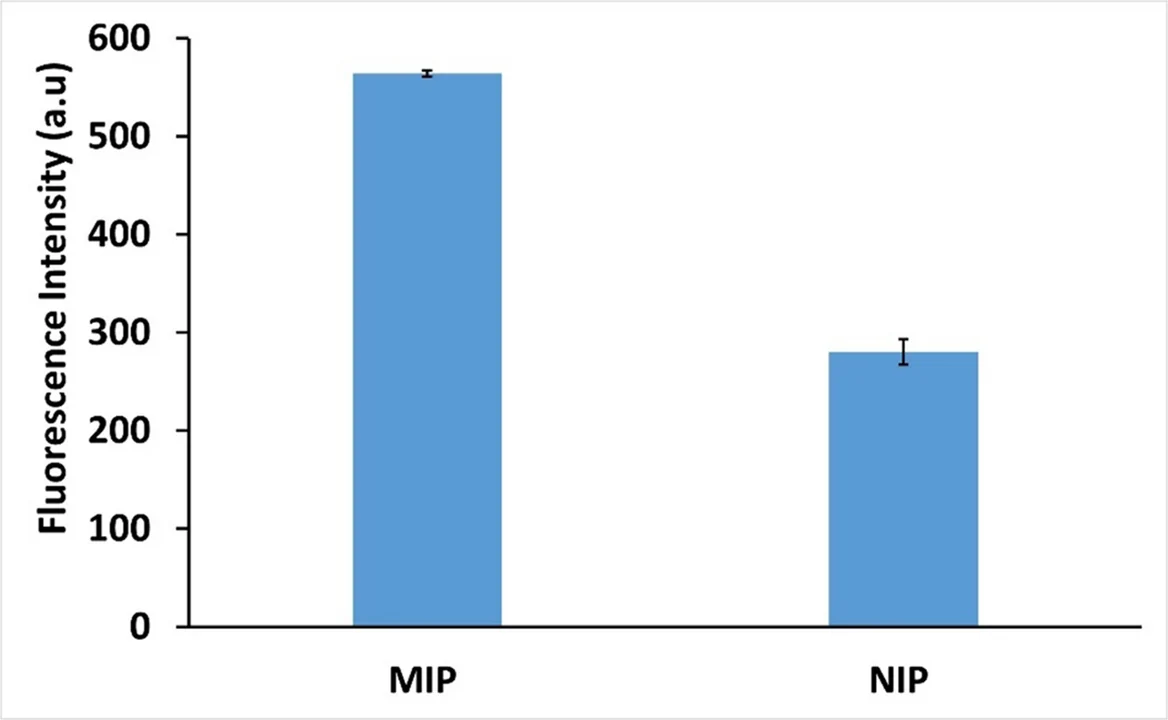
Comparison of the fluorescence responses of MIP and NIP thin-films following exposure to 40 µg L⁻^1^ DBT under identical experimental conditions (*n* = 3). Error bars represent ± SD

To verify that the increased fluorescence response was based on molecularly imprinting rather than non-selective adsorption onto the surface of the polymer, fluorescence responses of both the MIP and NIP are shown for the same DBT solution concentrations (40 µg L⁻^1^) as indicated in Fig. 2. The fluorescence intensity measured for the MIP was higher than that of the NIP where non-selective interaction occurred only at the surface of the NIP; this clearly indicates successful formation of highly specific recognition cavities in the MIP. Therefore, it can be concluded that much larger amounts of DBT were bound into the recognition sites within the MIP. Although fluorescence intensity does not contribute to the determination of the IF, the large difference in fluorescence intensities between the MIP and NIP supports the concept that the MIP has a greater binding capacity and therefore demonstrates molecular recognition properties similar to those indicated by the IF values reported in Table 1. Thus, the improved analytical performance of the new material is based upon its ability to recognize molecules selectively rather than through non-selective adsorption.

The reusability of the MIP thin-film was assessed by regenerating the films after each measurement cycle, via a hexane wash, followed by a methanol wash to release the previously bound DBT. The regenerated films were then tested again in successive adsorption-fluorescence measurements. Three regeneration cycles were carried out before a decrease in sensitivity was noticed, suggesting some degree of loss in binding capacity or recovery of the original imprinting site upon repeated regeneration. While regeneration was not considered essential for practical purposes due to both the low material costs associated with the production of the MIP thin-film and the fact that only an extremely small quantity of polymer was employed during film formation, the data obtained do provide evidence that the MIP thin-films have reasonable short-term reuse capabilities with good reproducibility and consistency of fluorescence response.

### Selectivity of DBT-MIP thin-film

**Fig. 3:**
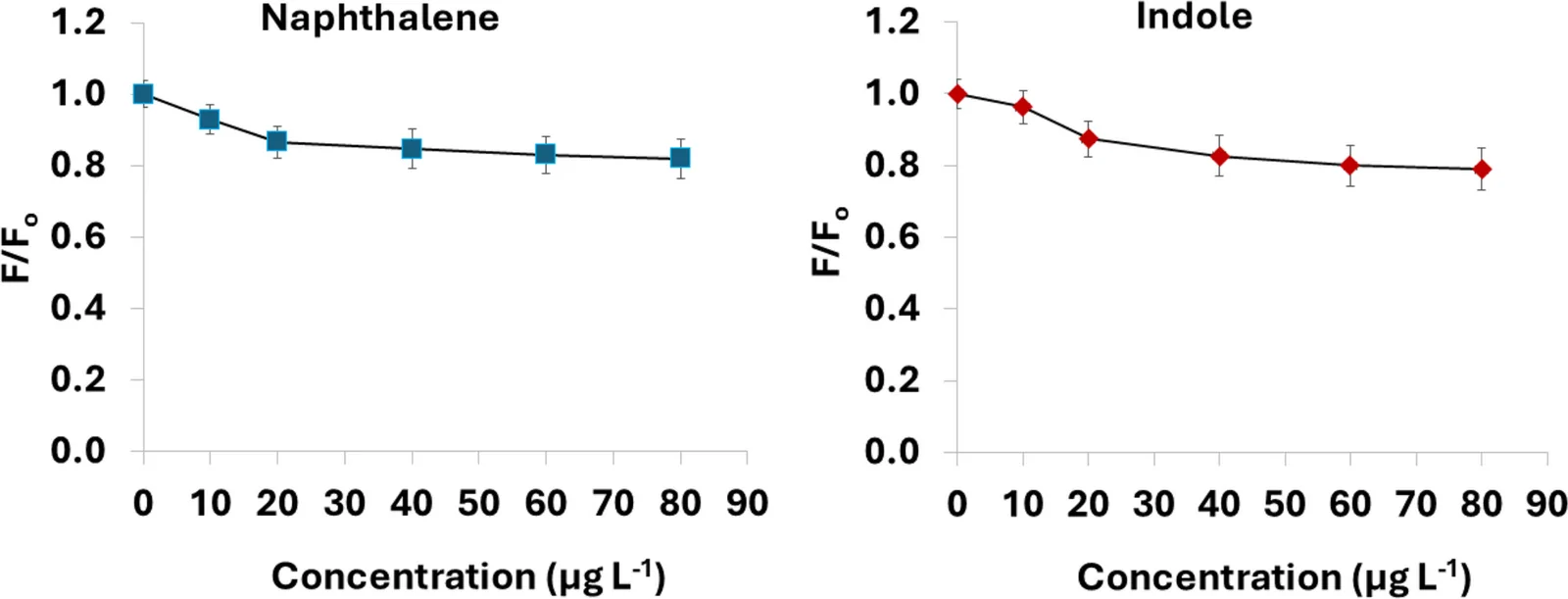
Selectivity of the MIP thin-film toward DBT in the presence of naphthalene and indole in DI water (0.0–80.0 µg L^−1^, *n* = 3), showing relative fluorescence response (F/F₀)

Selectivity tests are very important to demonstrate the reliability of MIP thin-film when used in real-world applications with large numbers of structurally related compounds, potentially interfering with analyte detection. The selectivity of the MIP thin-film toward DBT was shown in our previous study through comparison of structurally similar aromatic compounds. However, this study investigated how real-world interferents affect the fluorescence signal of DBT under relevant conditions. Specifically, the two aromatic interferants, naphthalene and indole, were chosen due to their common occurrence in marine environments to determine if they affected the fluorescence response of the MIP thin-film and to verify that reliable DBT quantitation could occur in various seawater matrices. As evidenced in Fig. 3, the fluorescence selectivity study shows that the MIP thin-film retains high recognition capabilities for DBT over all ranges of interferant concentrations tested (0.0–80.0 µg L^−1^) in the presence of aromatic interferants (naphthalene and indole) present in seawater. While there is evidence of moderate competitive effects occurring as the interferant concentration increases (as indicated by decreasing relative fluorescence intensities), the MIP thin-film preserves its selective binding in the imprinted cavities. For example, in the presence of naphthalene, the relative fluorescence intensity (F/F₀) decreased from 1.00 at 0 µg/L to 0.93, 0.86, 0.84, 0.83 and 0.82 at 10.0, 20.0, 40.0, 60.0 and 80.0 µg/L, respectively, corresponding to a total signal loss of approximately 18% throughout the evaluated concentration range. As noted in the results section, the decrease followed by a plateau-like region at high concentrations indicates that naphthalene primarily interacts via weak π–π stacking interactions with little capacity to interact competitively for DBT’s unique imprinted cavities. A slightly larger interference effect is observed when evaluating the influence of indole on the fluorescence response of the MIP thin-film. In this case, F/F₀ decreased from 1.00 at 0 µg L^−1^ to 0.96, 0.87, 0.82, 0.80 and 0.79 at 10.0, 20.0, 40.0, 60.0 and 80.0 µg L^−1^, respectively, corresponding to an approximate total signal loss of 21%. A greater signal loss than observed with naphthalene likely occurs because indole’s heterocycle includes a nitrogen atom that enables it to form both hydrogen bonding and dipole–dipole interactions with functional groups in the polymer matrix, in addition to π–π interactions. Thus, these additional types of interaction create an increased affinity of indole for areas of the polymer surface outside of DBT’s unique imprinted cavities, resulting in moderate levels of competition for DBT at higher concentrations.

### Real sample application

**Fig. 4:**
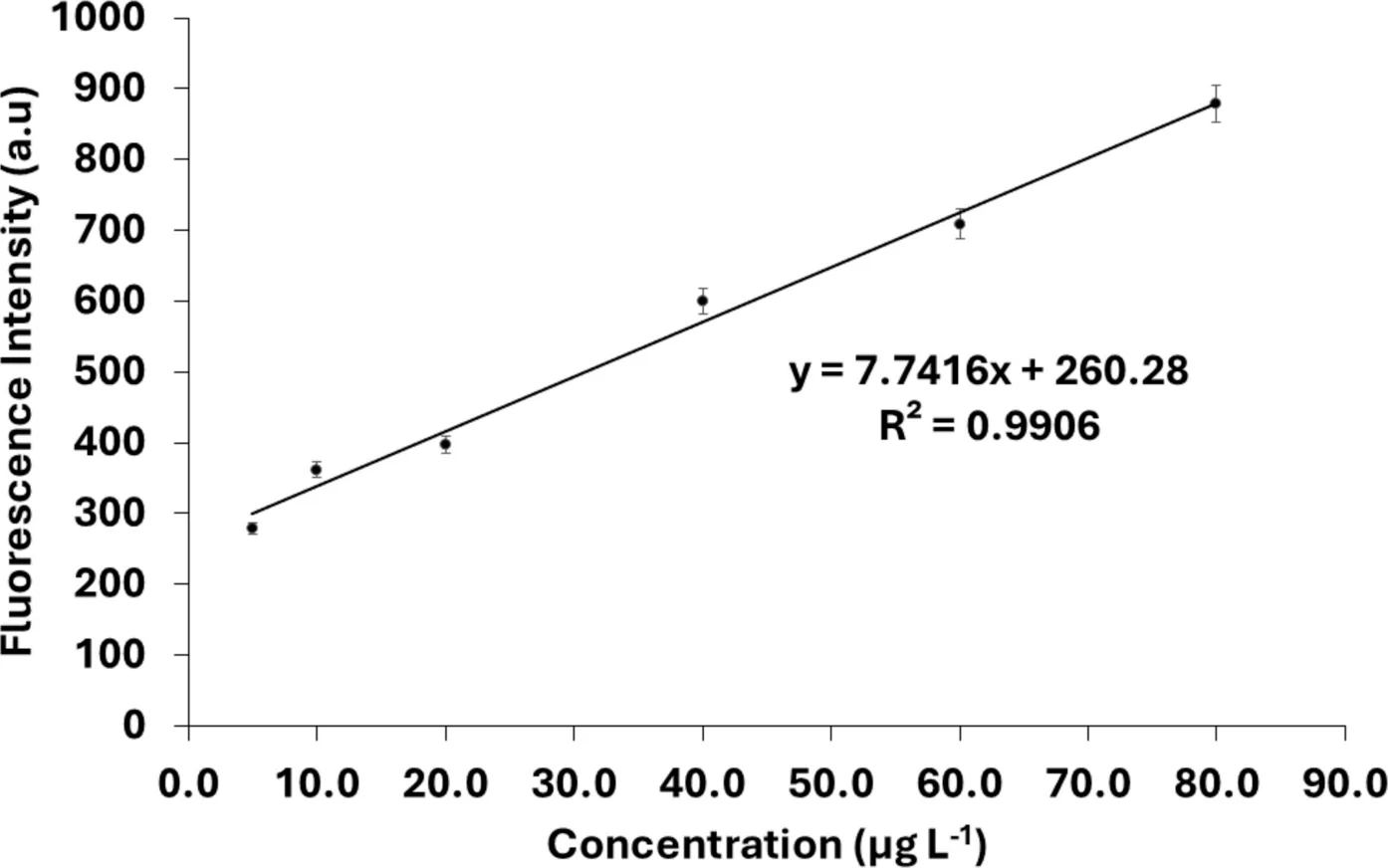
Calibration curves of the analysis of DBT compound by MIP thin-film-fluorescence in seawater samples in a range of 5–80 μg L^−1^ (RSD = 5.7%, *n* = 3)

To determine whether the developed MIP thin-film fluorescence method could be applied selectively to extract and measure DBT in real-world environmental samples, the tests were carried out on natural seawater sampled from Doha harbor. In order to simulate “realistic” test conditions, unfiltered seawater samples were spiked at various concentrations (5.0 to 80.0 µg L^−1^) while keeping the composition of the seawater as close to normal as possible. The seawater samples had been previously spiked with DBT. The data illustrated by the calibration curve in Fig. 4 showed a linear relationship between the fluorescence measured from the MIP thin-films and the DBT concentrations in the seawater samples (calculated *R*^2^ value = 0.9906; calculated slope = 7.7416; calculated y-intercept = 260.8). This demonstrates that the fluorescence produced by the MIP thin-film is proportional to the amount of DBT adsorbed into the imprinted sites, regardless of what else might be present in the seawater. While there is an obvious nonzero y-intercept (262.72), this represents the contribution of the background noise from both the polymer matrix itself and other residual signals that appear when no DBT has been spiked. Since this is consistent from sample to sample, it allows for accurate quantitation. That a gradient in fluorescence signal occurred as DBT concentration increased shows that the MIP thin-film retains both its selectivity and capacity to bind DBT in samples containing actual seawater.

The limit of detection (LOD) of this analysis method was defined by the relative standard deviation (5.7%) of a blank sample analyzed multiple times (*n* = 15). Additionally, the LOD was defined based on the slope from the calibration curve using the 3σ/slope criteria and the LOD was determined to be 1.74 μg/L. As stated above, the relatively low LOD value reflects the good sensitivity of this method and thus validates its use for determining trace amounts of DBT in unfiltered seawater samples, including those containing many complex matrix components. Furthermore, the calibration data also shows that no measurable amount of DBT could be detected in seawater that had been sampled without spiking with DBT. Therefore, it can be concluded that no detectable petroleum contamination occurred in the seawater samples collected under normal environmental conditions. Therefore, the MIP thin-film fluorescence method presented here is well-suited to monitor DBT when it has been added to the marine environment, e.g., through an oil spill event. Because the MIP thin-film continues to function successfully in undiluted seawater, this clearly shows one major benefit of using an imprint-based approach for recognizing DBT: namely, it provides specific binding to DBT over all other molecules that may be present in solution. The continued linearity and sensitivity demonstrated here is further evidence that nonspecific binding is minimized and/or eliminated due to matrix effects being suppressed. Additionally, since direct solid-state fluorescence measurements can be made without prior extraction or extensive sample processing steps, this greatly decreases both the number of required procedural steps and the overall time required to analyze each sample.

Compared to the DI water calibration curve, a comparative study of calibration curves obtained in DI water and in seawater (*n* = 3) using DBT concentrations ranging from 5.0–80.0 μg L^−1^ revealed that the fluorescence performance of the MIP thin-film remained stable in both media. However, some matrix effects were observed. Using DI water resulted in a linear relationship with a slope of 6.7231 and *R*^2^ = 0.9943, whereas using seawater resulted in a slightly greater slope of 7.7491 and *R*^2^ = 0.9906. It should be noted that this greater slope indicates greater sensitivity to fluorescence signals in seawater than in DI water.

The reason for this increase in sensitivity can be attributed to the higher ionic strength of seawater. The salting-out effect has an important influence on how hydrophobic organic compounds behave when there are high levels of dissolved salt present. As the concentration of dissolved ions increases, there is a preference for water to be associated with the ions, and thus the structure of the hydrogen-bonded water molecule arrangement becomes more ordered, which reduces the ability of water to associate with the hydrophobic components (Long & McDevit, 2002; Xie et al., 1997). Thus, as the amount of salt added to the solution increases, the solubility of hydrophobic compounds such as DBT decreases and therefore increases the tendency for DBT to be transferred out of the aqueous phase and into hydrophobic areas within the polymer matrix, i.e. the cavities created by imprinting. These increased hydrophobic interactions between DBT and the MIP result in an increase in the extent of uptake of DBT by the polymer and, consequently, an increase in what can be considered the apparent sensitivity of the system to DBT in saline media. Additionally, dissolved ions can inhibit non-specific binding of analytes and stabilize the local microenvironment around individual analyte molecules within the polymer matrix, thereby influencing fluorescence signals more positively. Although the seawater matrix is very complex compared to DI water, it did not significantly affect the linearity of fluorescence responses, and therefore, the matrix effect was minor. Seawater samples were analyzed in their natural state, without pH adjustment, to reflect real-world conditions that may be encountered during environmental monitoring. Since DBT is a neutral aromatic sulfur compound and does not ionize in the typical pH range found in seawater (approximately pH 7.7–8.3), the molecule’s chemical structure and charge remain almost entirely unaltered. A further study to assess thin-film performance for use as a sensor at a broader pH scale will be the subject of future investigations.

The proposed method was compared with other recognized analytical methods (Table 2), highlighting the compromises made between sensitivity, selectivity and applicable practicality when detecting aromatic hydrocarbon pollutants in aquatic environments. GC–MS and GC–MS/MS (Peng et al., 2023; Valera-Tarifa et al., 2018) are still the most sensitive and selective methods available for the detection of petroleum-related compounds with an excellent compound-specific identification capability, although it requires extensive sample preparation, including extraction, purification and concentration, before analysis with sophisticated instrumentation requiring highly trained personnel. In comparison, Fluorescence Spectroscopy can be analyzed quickly with an easy-to-use format; however, its applicability is often limited by low selectivity due to overlapping spectra and interference from naturally occurring organic matter present in marine matrices. MIP-based sensor platforms (Belbruno, 2018; Gkika et al., 2024) offer improved selectivity through the introduction of recognition sites that selectively recognize target molecules while retaining relatively straightforward analytical formats. Even though there is improved selectivity when compared to traditional optical methods, most of the current MIP platforms are used as selective extraction/pre concentration materials, requiring additional analysis after recovery of analytes. Therefore, the total analytical performance of MIP platforms is dependent upon both the molecular recognition and the ability to recover the analyte and the subsequent detection technique. Previously reported MIP-based fluorescent thin film sensor technologies (Chen et al., 2004; Wei et al., 2015) integrated molecular recognition with fluorescence sensing, thus enabling a simpler analytical workflow than that provided by conventional extraction-based MIP technologies. However, these systems have been designed to be operated in the laboratory under well-defined conditions and are therefore not optimized for the direct analysis of untreated seawater. The conventional fluorescence spectroscopy methods listed in Table 2 (Pärt et al., 2021; Poudel et al., 2025) were generally designed to screen for total petroleum contamination and were not specifically designed to selectively identify each of the individual compounds that could include DBT and therefore could increase spectral overlap and reduce selectivity. Rather than competing with chromatographic GC–MS laboratory-based methods in terms of detection limits, the proposed PET-supported MIP thin-film addresses the need for fast environmental screening with minimal sample preparation. While having higher detection limits (1.74 µg L⁻^1^) than chromatographic methods, it offers selectivity in recognizing DBT along with direct solid-state fluorescence measurements, eliminating extraction steps and reducing total analysis time. In addition, a PET substrate was used as an additional enhancement for making the sensing platform more practical in offering lightweight, low-cost, mechanical stability, and flexibility for the MIP thin-film; this will allow for easy fabrication and handling and still maintain consistent analytical results. As a whole, combining selective molecular recognition and direct optical detection (solid-state) of DBT, along with minimizing sample preparation and the practical benefits of being able to fabricate on a PET substrate, offers the potential to make this method suitable for routine environmental monitoring and provides a strong foundation for developing field-deployable sensing systems in the future.

**Table 2:** Comparison of analytical methods for the detection of DBT/aromatic hydrocarbons

Method	Limit of Detection (LOD)	Analysis Time	Selectivity	References
GC–MS/GC–MS/MS	~ 1–3 ng L⁻^1^	Hours (sample prep & analysis)	Excellent (compound-specific identification)	(Peng et al., 2023; Valera-Tarifa et al., 2018)
Fluorescence spectroscopy (bulk seawater monitoring)	~ 1–10 µg L⁻^1^	Minutes	Low–moderate (matrix interference)	(Pärt et al., 2021)
Fluorescence spectroscopy (marine monitoring)	~ 1.0–10 µg L⁻^1^	Minutes	Low–moderate (matrix interference)	(Poudel et al., 2025)
MIP-based sensors (general)	~ 0.1–5 µg L⁻^1^	Minutes to hours	High (template-specific binding)	(Belbruno, 2018; Gkika et al., 2024)
MIP thin-film fluorescence (literature)	~ 1–10 µg L⁻^1^	Minutes	High	(Chen et al., 2004; Wei et al., 2015)
This work (MIP thin-film + solid-state fluorescence)	1.74 µg L⁻^1^	~ 1 h (no extraction)	High (selective toward DBT)	This study

## Conclusions

This research has demonstrated the development of a thin-film-based fluorometric assay using an MIP for the specific determination of DBT as an indicator of oil contamination in seawater. The MIP thin-films were stable mechanically when placed onto a PET substrate without surface modifications; thus, they could be fabricated and manipulated simply. The use of low-fluorescence polymer components enabled direct solid-state fluorescence measurements without analyte extraction. Due to the effective adsorption of DBT by the imprinted cavities, the MIP-based system produced clear concentration-dependent fluorescence responses over the range of 5.0–80.0 µg L^−1^, with good linearity (*R*^2^ = 0.9906), in both deionized water and seawater matrices. With a low limit of detection (LOD) of 1.74 µg L^−1^, the method also displayed a very good level of selectivity towards DBT in the presence of structural analogs of aromatic compounds such as naphthalene and indole, albeit at levels of minimal signal suppression.

These results show clearly that the imprinted cavities were able to selectively recognize DBT, thereby producing reliable results even in complex matrices, demonstrating applicability to real seawater samples. Additionally, since there was little to no matrix effect observed in this research, and where possible, increased sensitivity due to increased ionic strength in these samples further indicates that this method can effectively function under realistic environmental conditions. Therefore, the combined features of fast adsorption, high selectivity, and acceptable sensitivity suggest that this method should be suitable for trace-level determinations of petroleum-related pollutants in marine systems. In addition, because the MIP thin-film adheres well to flexible PET substrate and allows for direct solid-state fluorescence measurement, it provides a basis for future sensor developments. In contrast to traditional MIP-based analytical systems, the suggested method allows fast fluorescence measurements from the MIP thin-film directly after contact with seawater. The simplicity of the described analytical process, along with its high degree of selectivity towards DBT and ability to operate without prior treatment of the sample, provides a basis for future sensor development for in-situ oil spills monitoring. While the prepared MIP thin-films showed acceptable mechanical strength throughout the polymerization step, methanol washing, removal of the template, and analysis of untreated seawater samples, the primary focus of this research was on demonstrating the analytical performance of the sensing method. Future research efforts will address the long-term durability of MIP thin-films in a marine environment for extended periods, as well as their regeneration efficiency and reuse efficiency through repeated adsorption–desorption cycles.

## Data Availability

All data supporting the findings of this study are included within the paper.
